# Thyroglobulin-to-tumor volume ratio combined with ultrasound features for diagnosing thyroid follicular neoplasms

**DOI:** 10.3389/fendo.2025.1626766

**Published:** 2025-07-10

**Authors:** Xixi Zhu, Fen Liu, Jiaye Liu, Zhihui Li, Yu Ma

**Affiliations:** ^1^ Division of Thyroid Surgery, Department of General Surgery; Laboratory of Thyroid and Parathyroid Diseases, Frontiers Science Center for Disease-Related Molecular Network, West China Hospital, Sichuan University, Chengdu, China; ^2^ Department of Respiratory and Critical Care Medicine, Frontiers Science Center for Disease-related Molecular Network, Center of Precision Medicine, Precision Medicine Key Laboratory of Sichuan Province, West China Hospital, Sichuan University, Chengdu, Sichuan, China; ^3^ Department of Operating Room, West China Hospital, West China School of Nursing, Sichuan University, Chengdu, Sichuan, China

**Keywords:** thyroid follicular neoplasm, follicular thyroid carcinoma, the serum thyroglobulin-to-tumor volume ratio, thyroglobulin, ultrasonography

## Abstract

**Objective:**

Current preoperative diagnostics inadequately differentiate benign from malignant thyroid follicular neoplasms. This study evaluated the diagnostic utility of thyroid function markers and contrast-enhanced ultrasound (CEUS) features in differentiating follicular thyroid adenoma (FTA) from follicular thyroid carcinoma (FTC), focusing on a novel parameter: the thyroglobulin-to-tumor volume ratio (Tg/Vol ratio).

**Methods:**

We retrospectively analyzed 432 resected thyroid follicular neoplasms. A comprehensive comparison was performed regarding baseline characteristics, thyroid function profiles, and CEUS features between FTA and FTC groups through univariate and multivariate binary logistic regression. Diagnostic performance was determined via receiver operating characteristic (ROC) curve analysis. The prevalence of FTC across serum marker subgroups was assessed, followed by the development of a multivariate diagnostic model integrating the Tg/Vol ratio with CEUS characteristics.

**Results:**

Among 432 patients (352 females, 81.5%) with a median age of 47 years, multivariate logistic regression analysis revealed three independent predictors of FTC: capsular involvement (odds ratio [OR] = 9.958, 95% confidence interval [CI]: 2.453 – 40.424, p = 0.001), Tg/Vol ratio >7.412 (OR = 3.508, 95% CI: 1.388 – 8.868, p = 0.008), and male gender (OR = 3.474, CI: 1.751 – 6.891, p < 0.001). Subgroup analyses revealed higher FTC prevalence in patients with Tg > 409.18 μg/L (20.41%, p = 0.002) and Tg/Vol ratio > 20.68 (20.41%, p = 0.009). The combined diagnostic model incorporating Tg/Vol ratio and CEUS features demonstrated 69.4% sensitivity, 77.0% specificity, and the area under the curve(AUC) of 0.769.

**Conclusion:**

While elevated preoperative Tg correlates with malignant potential, but the Tg/Vol ratio emerges as a more robust preoperative discriminator. The combined diagnostic model incorporating Tg/Vol ratio and CEUS features significantly improves FTC detection accuracy.

## Introduction

Thyroid follicular neoplasms, the second most prevalent thyroid tumors, are histologically classified into follicular thyroid adenoma (FTA) and follicular thyroid carcinoma (FTC) (1). The diagnostic gold standard—identification of capsular and/or vascular invasion—can only be confirmed postoperatively through pathological examination, posing a critical clinical dilemma in preoperative decision-making (2).

While ultrasonography serves as the primary screening modality for thyroid nodules, its diagnostic accuracy for follicular neoplasms remains suboptimal (3, 4). The European Thyroid Association guidelines noted that FTC may more frequently exhibit irregular margins and microcalcifications (5); however, a recent study involving 705 patients revealed significant limitations in conventional Thyroid Imaging Reporting and Data System in differentiating FTC from FTA (6). Unlike papillary thyroid carcinoma (PTC), fine-needle aspiration biopsy demonstrates limited diagnostic value for FTC due to its inability to assess capsular or vascular invasion, the pathological hallmarks of malignancy (7).

In contrast to medullary thyroid carcinoma (MTC), where calcitonin serves as a reliable serum marker (8), no validated preoperative biomarker currently exists for FTC (9). Emerging evidence suggests that serum thyroglobulin (Tg) levels correlate with both the secretory activity and malignant potential of thyroid tumors, with FTC typically exhibiting elevated Tg secretion (10, 11). This positions Tg as a potential discriminator between benign and malignant follicular neoplasms.

However, serum Tg concentration alone suffers from limited specificity due to confounding factors, including thyroid volume and inflammatory status (12, 13). While Tg levels exhibit nonspecific elevation due to thyroid tissue volume, the nonlinear correlation between tumor size and neoplastic Tg production further compromises diagnostic accuracy. (14). To address these limitations, the serum thyroglobulin-to-tumor volume ratio (Tg/Vol ratio), a novel parameter correcting for tumor dimension, may better reflect the functional-morphological disparities between FTA and FTC by normalizing secretory output to lesion size.

Therefore, this study aimed to evaluate the diagnostic utility of preoperative thyroid function profiles and CEUS features in distinguishing FTA from FTC; and develop a composite diagnostic model to enhance preoperative FTC detection; Additionally, this represents the first large-scale study to systematically evaluate the diagnostic efficacy of the noninvasive Tg/Vol ratio as a novel discriminator for thyroid follicular neoplasms.

## Materials and methods

### Study population

This retrospective study analyzed consecutive patients who underwent initial surgical resection for thyroid follicular neoplasms at West China Hospital between January 2012 and December 2024. Exclusion criteria were as follows: (1) Insufficient preoperative data (serum thyroid markers or CEUS examinations); (2) Final pathological diagnosis of follicular tumor of uncertain malignant potential (FT-UMP) or noninvasive follicular thyroid neoplasm with papillary-like nuclear features (NIFTP); (3) Concurrent acute or chronic thyroiditis (Patients with clinical manifestations of neck pain, swelling, or tenderness, positive thyroid autoantibodies, and characteristic ultrasonographic features such as heterogeneous parenchymal echotexture or increased vascularity); (4) Predominantly cystic lesions or significant thyroid gland enlargement. The study was assessed and approved by the ethics committees of the hospitals (No.20242251). The requirement for written informed consent was waived due to the study’s retrospective design and no patients were excluded based on consent status.

### Serological analysis

Serum thyroglobulin (Tg; reference range: 3.50 – 77.00 μg/L), thyroglobulin antibodies (TgAb; 0 – 115.00 IU/mL), thyroid peroxidase antibodies (TPOAb; 0 – 34.00 IU/mL), and thyroid-stimulating hormone receptor antibodies (TRAb; 0 – 1.75 IU/mL) were quantified via electrochemiluminescence immunoassay. Thyroid-stimulating hormone (TSH; 0.27 – 4.2 mIU/L), free triiodothyronine (FT3; 3.60 – 7.50 pmol/L), and free thyroxine (FT4; 12.0 – 22.0 pmol/L) levels were measured using chemiluminescent immunoassay.

TgAb positivity was defined as ≥ 115.00 IU/mL. Given potential TgAb interference, Tg analysis was restricted to TgAb-negative patients (< 115.00 IU/mL). For Tg values exceeding the upper detection limit (5,000 μg/L), results were censored at 5,000 μg/L. All measurements were conducted in this center according to unified standard protocols to ensure reproducibility.

### Tumor volume calculation

Tumor volume (cm³) was calculated using the prolate ellipsoid formula, which is currently one of the standard methods for ultrasonographic tumor volume measurement and has been validated in prior thyroid studies (15). Dimensions were obtained from CEUS reports, with data validity confirmed through rigorous reliability testing.


$$Volume=\left(\pi /6\right)\times \;length\times width\times height$$

### Image analysis

A linear array probe (5–12 MHz) was used. Patients assumed a supine position with full neck exposure. Contrast-enhanced ultrasound dynamically visualized multiple thyroid sections. Two experienced sonographers independently analyzed all image parameters; discrepancies were resolved by a third physician’s consultation for consensus.

### Statistical analysis

All statistical analyses were performed using SPSS 27.0 (IBM Corp., Armonk, NY, USA), with a two-tailed significance level set at α = 0.05. Continuous variables with non-normal distributions were expressed as median (range) and compared using the Mann-Whitney U test. Categorical variables were presented as frequencies (percentages) and analyzed by χ² test or Fisher’s exact test, as appropriate.

To identify risk factors for FTC, both univariate and multivariate binary logistic regression analyses were conducted. The diagnostic performance of significant predictors was evaluated using receiver operating characteristic (ROC) curve analysis, with sensitivity and specificity reported. For statistically significant indicators, the area under the ROC curve (AUC) was compared using DeLong’s test to assess predictive value and determine optimal cutoff thresholds. A combined diagnostic model was subsequently developed through binary logistic regression.

Additionally, Spearman’s rank correlation analysis was employed to examine the association between FTC prevalence and various patient subgroups, with correlation coefficients (r) reported.

## Results

### Clinical characteristics

The study cohort comprised 432 patients with thyroid follicular neoplasms, including 377 cases of FTA and 55 cases of FTC. The cohort included 352 females (81.5%) and 80 males (18.5%), with an age range of 3–80 years (mean ± standard deviation [SD], 47.4 ± 13.6 years).

Comparative analysis between FTA and FTC groups revealed statistically significant differences in male predominance (15.1% vs. 41.8%, p < 0.001) and preoperative serum FT3 levels (median [interquartile range, IQR]: 4.75 [4.32 – 5.32] vs. 5.06 [4.54 – 5.58] pmol/L, p = 0.033). No significant differences were observed in age, body mass index (BMI), maximal tumor diameter, tumor volume, or levels of TgAb, TPOAb, TRAb, TSH, or FT4. Additionally, the proportions of patients with elevated TgAb, TPOAb, or TRAb did not differ significantly between groups (**Table 1**).

**Table 1:** Comparison of clinical characteristics between FTA and FTC patients.

Characteristic	FTA (n = 377)		FTC (n = 55)		p-value
Male	57	(15.1%)	23	(41.8%)	< 0.001^*^
Age (years)	48	(39 – 57)	47	(34 – 58)	0.496
BMI	22.83	(20.73 – 24.97)	23.67	(21.49 – 26.72)	0.091
Maximum tumor diameter (cm)	4.60	(3.60 – 5.70)	4.40	(3.80 – 5.70)	0.970
Tumor volume (cm³)	18.35	(9.67 – 37.27)	21.22	(11.47 – 38.61)	0.354
TgAb (IU/mL)	15.70	(13.40 – 19.35)	15.70	(13.30 – 33.10)	0.756
TgAb > 115 IU/mL	34	(9.0%)	6	(10.9%)	0.651
TPOAb (IU/L)	9.11	(9.00 – 16.45)	9.42	(9.00 – 15.50)	0.859
TPOAb > 34.00 IU/L	55	(14.6%)	7	(12.7%)	0.713
TRAb (IU/L)	0.80	(0.80 – 0.80)	0.80	(0.80 – 0.87)	0.118
TRAb > 1.75 IU/L	8	(2.1%)	2	(3.6%)	0.828
TSH (mIU/L)	1.89	(1.19 – 2.90)	1.78	(1.10 – 2.84)	0.810
FT3 (pmol/L)	4.75	(4.32 – 5.32)	5.06	(4.54 – 5.58)	0.033^*^
FT4 (pmol/L)	15.00	(13.40 – 16.80)	14.80	(13.40 – 16.20)	0.526

FTA, Follicular thyroid adenoma; FTC, Follicular thyroid carcinoma; TgAb, Thyroglobulin antibody; TPOAb, Thyroid peroxidase antibody; TRAb, Thyrotropin receptor antibody; TSH, Thyroid-stimulating hormone; FT3, Free triiodothyronine; FT4, Free thyroxine

^*^p-value < 0.05.

To minimize interference from elevated TgAb, analyses involving Tg and Tg/Vol ratio were restricted to TgAb-negative patients (< 115.00 IU/mL). Among 392 TgAb-negative patients (343 FTA, 49 FTC), significant intergroup differences were noted in preoperative serum Tg levels (122.00 [40.10 – 345.00] vs. 271.00 [137.00 – 962.00] μg/L, p < 0.001), Tg elevation prevalence (62.7% vs. 77.6%, p = 0.042), and Tg/Vol ratio (6.98 [2.86 – 18.54] vs. 13.02 [7.63 – 51.71], p < 0.001). No differences were detected in maximal tumor diameter, tumor volume, or TgAb levels (**Table 2**).

**Table 2:** Comparison of clinical characteristics between TgAb-negative FTA and FTC patients.

Characteristic	FTA (n = 343)		FTC (n = 49)		p-value
Maximum tumor diameter (cm)	4.60	(3.70 – 5.70)	4.40	(3.80 – 5.75)	0.952
Tumor volume (cm³)	18.39	(9.95 – 37.64)	21.22	(11.30 – 38.77)	0.366
Tg (ug/L)	122.00	(40.10 – 345.00)	271.00	(137.00 – 962.00)	< 0.001^*^
Tg > 77 ug/L	215	(62.7%)	38	(77.6%)	0.042^*^
TgAb (IU/ml)	15.30	(13.30 – 17.90)	14.80	(13.20 – 20.85)	0.888
Tg/Vol ratio	6.98	(2.86 – 18.54)	13.02	(7.63 – 51.71)	< 0.001^*^

FTA, Follicular thyroid adenoma; FTC, Follicular thyroid carcinoma; Tg, Thyroglobulin; TgAb, Thyroglobulin antibody.

^*^p-value < 0.05.

### Ultrasonographic features

Comparative analysis of 377 FTA and 55 FTC cases demonstrated significant differences in tumor morphology (p = 0.003), margin clarity (p = 0.020), calcification patterns (p < 0.001), and capsular involvement (p < 0.001). No significant variations were observed in tumor location (p = 0.158), aspect ratio (p = 1.000), cystic-solid composition (p = 0.172), or Adler blood flow grading (p = 0.773) (**Supplementary Table 1**).

### Odds ratios of clinical and ultrasonographic characteristics

Univariate analysis revealed statistically significant differences between FTA and FTC patients regarding gender, FT3 levels, Tg levels, Tg/Vol ratio, tumor morphology, margin clarity, presence and type of calcifications, and capsular involvement (all p < 0.05).

**Figure 1:**
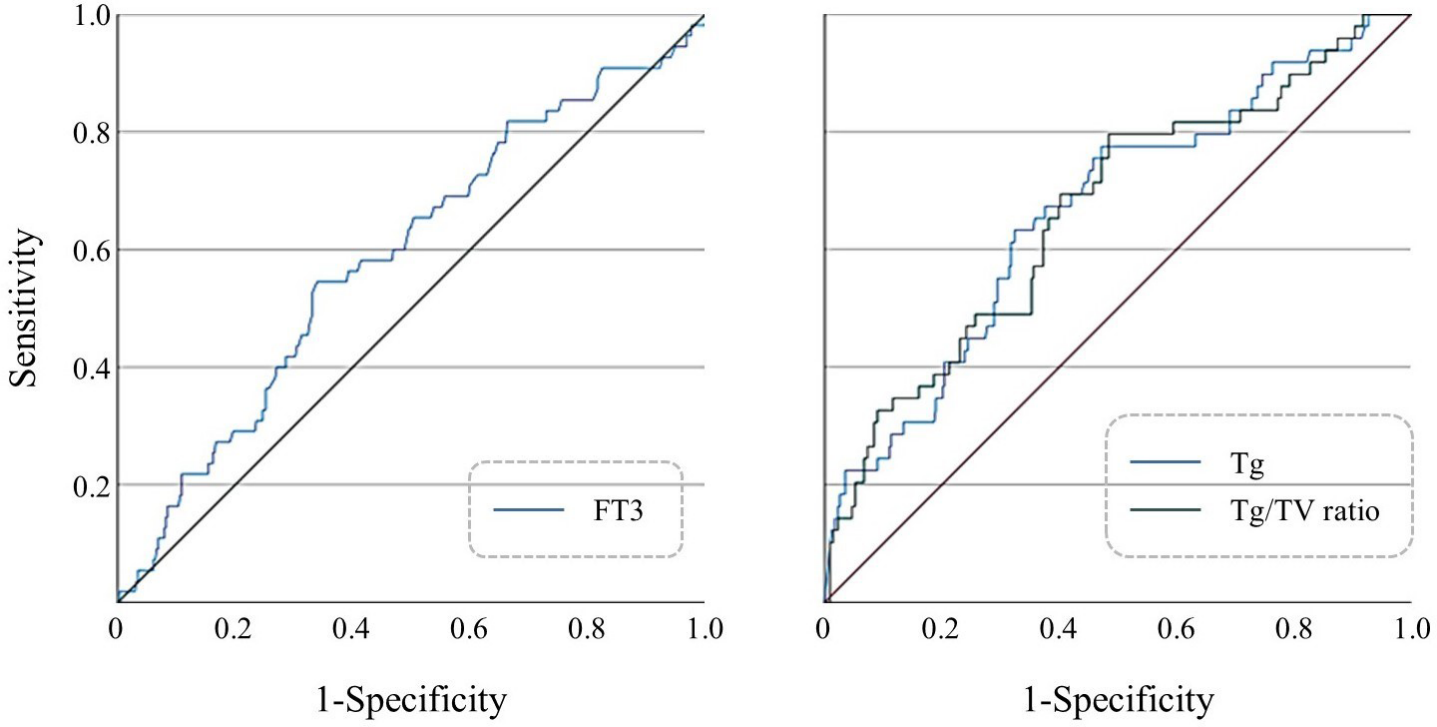
(Left) ROC curve of preoperative FT3 levels for predicting FTC; (Right) ROC curves of preoperative serum Tg levels and Tg/Vol ratio for predicting FTC.

ROC curves were constructed for FT3, Tg, and Tg/Vol ratio in FTC diagnosis (**Figure 1**). Optimal cutoff values determined by Youden’s index were: FT3 > 5.045 pmol/L (AUC 0.647), Tg > 217.5 μg/L (AUC 0.608), and Tg/Vol ratio > 7.412 (AUC 0.631) (**Supplementary Table 2**).

### Diagnostic logistic regression analysis

Variables demonstrating statistical significance in univariate analysis (male gender, FT3 > 5.045 pmol/L, irregular tumor shape, irregular margins, microcalcifications, and capsular involvement) were subsequently incorporated into multivariate logistic regression modeling (**Supplementary Table 3**). The analysis revealed male gender (p < 0.001) and capsular involvement (p = 0.001) as statistically significant independent risk factors, while FT3 > 5.045 pmol/L (p = 0.105), irregular tumor morphology (p = 0.377), irregular margins (p = 0.681), and microcalcifications (p = 0.297) failed to demonstrate independent predictive value.

To mitigate potential confounding effects from thyroglobulin antibodies (TgAb), analyses incorporating Tg and Tg/Vol ratio were exclusively performed in TgAb-negative patients (<115.00 IU/mL, n=392). Multivariate evaluation of significant univariate predictors (Tg > 217.5 μg/L and Tg/Vol ratio > 7.412) identified only Tg/Vol ratio > 7.412 (p = 0.008) as maintaining independent predictive significance, whereas Tg > 217.5 μg/L (p = 0.507) showed no statistically meaningful association (**Table 3**).

**Table 3:** Binary logistic regression analysis of clinical characteristics and CEUS features in predicting FTC in TgAb-negative patients ^a^.

Characteristic	FTA (n = 343)		FTC (n = 49)		Univariate Logistic Regression			Multivariate Logistic Regression		
					OR	(95% CI)	p-value	OR	(95% CI)	p-value
Maximum tumor diameter (cm)	4.60	(3.70 – 5.70)	4.40	(3.80 – 5.75)	1.011	(0.842 – 1.214)	0.908			
Tumor volume (cm³)	18.39	(9.95 – 37.64)	21.22	(11.30 – 38.77)	1.001	(0.994 – 1.009)	0.712			
Tg (ug/L) ^b^										
≤ 77.00	128	(37.3%)	11	(22.4%)	1.000		–	1.000		–
77.00 – 217.50	104	(30.3%)	7	(14.3%)	0.783	(0.293 – 2.092)	0.626	0.457	(0.155 – 1.344)	0.155
217.50	111	(32.4%)	31	(63.3%)	3.250	(1.561 – 6.766)	0.002^*^	1.376	(0.536 – 3.531)	0.507
TgAb (IU/ml) ^c^										
≤ 13.30	81	(23.6%)	15	(27.3%)	1.000		–			
13.30 – 15.30	88	(25.7%)	7	(27.3%)	0.898	(0.397 – 2.028)	0.795			
15.30 – 18.10	82	(23.9%)	12	(10.9%)	0.488	(0.190 – 1.253)	0.136			
> 18.10	92	(26.8%)	15	(34.5%)	1.136	(0.523 – 2.467)	0.748			
Tg/Vol ratio ^d^										
≤ 7.412	177	(51.6%)	10	(20.4%)	1.000		–	1.000		–
> 7.412	166	(48.4%)	39	(79.6%)	4.158	(2.011 – 8.597)	< 0.001^*^	3.508	(1.388 – 8.868)	0.008^*^

FTA, Follicular thyroid adenoma; FTC, Follicular thyroid carcinoma; Tg, Thyroglobulin; TgAb, Thyroglobulin antibody; OR, Odds ratio; CI, Confidence interval

a Due to potential interference from elevated TgAb, analyses involving Tg and Tg/Vol ratio were performed only in TgAb-negative patients (< 115.00 IU/mL).

b In the Tg subgroup, patients were divided into 3 groups based on the upper limit of normal preoperative Tg value (77 ug/L) and the optimal cutoff value (217.5 ug/L) derived from univariate analysis.

c All patients were divided into 4 groups using the quartile method.

d In the Tg/Vol ratio subgroup, patients were divided into 2 groups using the optimal cutoff value (7.412) derived from univariate analysis.

^*^p-value < 0.05.

The final multivariate predictive model established three independent risk factors for follicular thyroid carcinoma, ranked in descending order of predictive strength: capsular involvement (adjusted odds ratio [OR] = 9.958; 95% CI: 2.453-40.424), Tg/Vol ratio >7.412 (adjusted OR = 3.508; 95% CI: 1.388-8.868), and male gender (adjusted OR = 3.474; 95% CI: 1.751-6.891).

### Prevalence of malignancy

Among all thyroglobulin antibody (TgAb)-negative patients, the overall prevalence of FTC was 12.5% (49/392). A statistically significant positive correlation was observed between malignancy rates and serum Tg concentrations (Pearson’s r = 0.176, p < 0.001). Notably, the subgroup with Tg levels exceeding 409.18 μg/L manifested a substantially elevated FTC prevalence (20/98 20.41%, p = 0.002) when compared to patients with lower Tg concentrations (**Supplementary Figure 1**).

Similarly, a proportional relationship was revealed between FTC prevalence and the Tg/Vol ratio (Pearson’s r = 0.155, p = 0.002). Patients exhibiting a Tg/Vol ratio surpassing 20.68 similarly demonstrated a significantly higher malignancy rate (20/98 20.41%, p = 0.009) relative to other ratio subgroups (**Supplementary Figure 2**).

### Diagnostic performance of multivariate model

**Figure 2:**
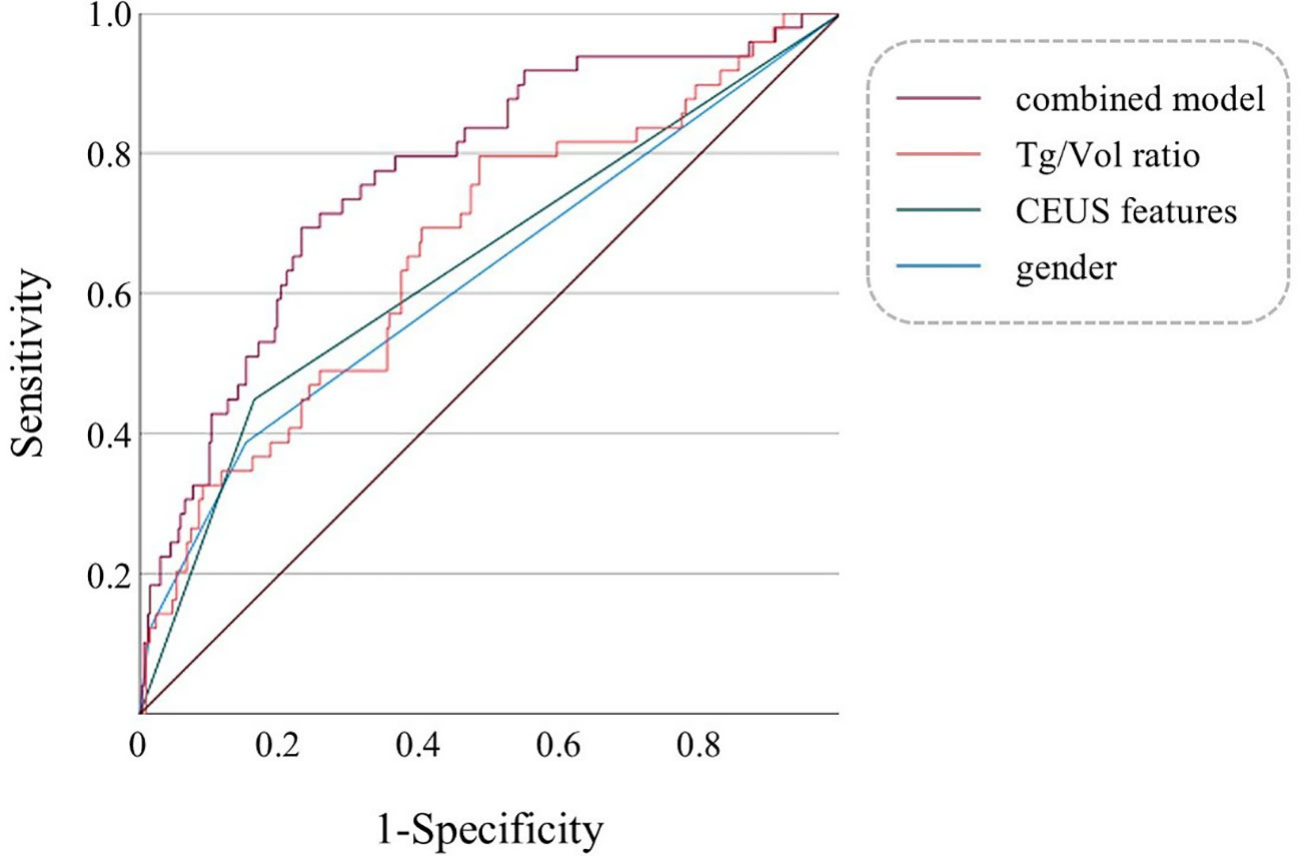
Multivariable ROC curves of gender, CEUS features, Tg/Vol ratio, and the combined model for diagnosing FTC. The combined model refers to a multifactorial diagnostic model incorporating gender, CEUS features, and the Tg/Vol ratio.

ROC curves were constructed to evaluate the diagnostic performance of individual parameters (gender, CEUS features, Tg/Vol ratio) and their combined multifactorial model for distinguishing FTC (**Figure 2**). Comparative analysis of the AUC revealed differential diagnostic efficacy among these approaches. The diagnostic value of CEUS features alone was marginally inferior to that of the Tg/Vol ratio alone. Notably, the multifactorial diagnostic model integrating Tg/Vol ratio with gender and CEUS features demonstrated superior performance (AUC 0.769) compared to any single-parameter approach, achieving 69.4% sensitivity and 77.0% specificity (**Table 4**). Decision curve analysis revealed the combined model conferred clinical utility in low-threshold ranges with maximum net benefit of 0.116, whereas the calibration curve demonstrated excellent probabilistic calibration (Brier=0.096). Notably, score distributions verified the model’s efficacy in segregating typical benign cases (**Supplementary Figures 3**-**5**).

**Table 4:** ROC curve analysis and diagnostic performance.

ROC curve	AUC	Cutoff Value	Sensitivity (%)	Specificity (%)	PPV (%)	NPV (%)
Gender	0.643 (0.553 – 0.733)	–	38.8	84.8	52.2	80.1
Ultrasonic features	0.625 (0.532 – 0.717)	–	44.9	83.7	54.1	78.0
Tg/Vol ratio	0.664 (0.581 – 0.748)	7.4129	79.6	51.6	41.3	45.4
Multifactorial diagnostic model	0.769 (0.698 – 0.841)	–	69.4	77.0	56.4	67.7

PPV, Positive predictive value; NPV, Negative predictive value.

## Discussion

To our knowledge, our study represented the first large-scale investigation to establish the clinical validity of the Tg/tumor volume ratio as a novel, noninvasive biomarker for preoperative discrimination between FTA and FTC. This readily calculable parameter addressed a critical unmet need in thyroid oncology by providing both diagnostic and prognostic information prior to surgical intervention.

The clinical management of follicular thyroid neoplasms remains challenging due to persistent diagnostic uncertainties in the preoperative phase (16, 17). Current practice patterns revealed two concerning trends: excessive surgical intervention for benign disease (18), as Ra et al. reported that the rate of unnecessary surgeries for resected follicular tumors was approximately 26% (19); and delayed diagnosis of malignant cases, with substantial proportion of misclassified FTC cases presenting with distant metastases at definitive diagnosis (20, 21). This diagnostic dilemma underscores the imperative for improved preoperative risk stratification tools.

The diagnostic value of serum Tg in preoperative differentiation of FTC remains controversial. While studies such as Chen et al. demonstrated correlations between serum Tg and FTC risk (10, 22, 23), inherent limitations persist. Recent studies reported that the existence of TgAb interferes with Tg measurement, compounded by the absence of standardized Tg thresholds (20 – 30% variability across immunoassay methods) (24, 25). These discrepancies suggest that although Tg reflects tumor secretory activity, its standalone diagnostic reliability remains suboptimal.

In TgAb-negative patients, our analysis revealed significantly higher preoperative serum Tg levels in FTC compared to FTA. However, multivariate analysis showed that Tg > 217.5 μg/L lost statistical significance when adjusted for the Tg/Vol ratio. This implies that large FTA may elevate Tg levels via mass effect leading to false positives, while small FTC could exhibit high Tg/Vol ratio despite modest absolute Tg concentrations due to early invasive potential. Consequently, the Tg/Vol ratio emerges as a more stable biomarker.

The optimal Tg/Vol ratio cutoff was 7.412 (AUC = 0.664, sensitivity 79.6%, specificity 51.6%), with multivariate validation confirming its independent predictive value (OR = 3.508, p = 0.008). Notably, FTC prevalence increased significantly when the Tg/Vol ratio exceeded 8.07 (p = 0.0092). These findings advocate incorporating preoperative Tg/Vol ratio into surgical decision-making and surveillance protocols.

While the Tg/Vol ratio is not an independent diagnostic tool, it serves as a valuable triage tool that significantly enhances the accuracy of risk stratification and refines the diagnostic evaluation of thyroid follicular neoplasms. For lesions with indeterminate fine-needle aspiration (FNA) results, a low Tg/Vol ratio may obviate unnecessary diagnostic resection surgery. Conversely, an elevated Tg/Vol ratio suggests increased malignant potential, necessitating prompt surgical consultation. Furthermore, given the limited sensitivity of intraoperative frozen section analysis for detecting thyroid follicular carcinoma, cases exhibiting elevated Tg/Vol ratios in conjunction with suspicious malignant ultrasound features warrant consideration for extended surgical intervention. Postoperatively, serial monitoring of the Tg/Vol ratio also aids in the early detection of tumor recurrence in patients who have undergone partial thyroidectomy.

Serum Tg levels are influenced by multifactorial physiological, pathological, and technical determinants. Emerging evidence highlights iodine nutritional status as a pivotal modulator of Tg, with both deficiency and excess inducing elevation. Additional influencers include TSH, thyroiditis, thyroid medications, and assay variations, all of which may perturb Tg levels and thereby impact Tg/Vol ratios. Thus, interpretation of Tg/Vol values requires consideration of these confounders, with trend monitoring offering greater diagnostic utility than single measurements.

Contrary to studies linking thyroid cancer with elevated TSH, FT3, or FT4 levels, our cohort (excluding patients with acute or chronic thyroiditis) showed no independent predictive value for these markers (26–28). Regarding imaging, conventional CEUS features (irregular morphology, irregular margins, microcalcifications, and capsular invasion) correlated with FTC. The 2015 American Thyroid Association (ATA) management guidelines corroborate our finding that capsular involvement demonstrated the highest predictive strength (OR = 9.958, specificity 83.7%), aligning with intraoperative frozen-section accuracy and underscoring its potential as a diagnostic gold standard (29).

The combined model integrating the Tg/Vol ratio with CEUS features achieved superior diagnostic balance (AUC = 0.769, representing a 12.1% improvement over individual metrics), with sensitivity 69.4% and specificity 77.0%. Suboptimal positive and negative predictive values may reflect our study’s relatively low FTC prevalence. Additionally, decision curve and calibration curve analyses showed the model excelled in predictive probability (Brier=0.096), low-threshold clinical value with the max net benefit of 11.6%, and typical benign case identification. Its main limitation was conservative malignant predictions, failing to reliably identify high-risk patients—attributed to dataset imbalance from insufficient positive cases. This highlights the need for future studies to collect more malignant cases for dataset balancing and develop risk stratification strategies accordingly.

Several limitations in our study should be mentioned. First, the single-center retrospective design precluded longitudinal assessment of thyroid functiondata. Second, while TgAb exclusion strengthened internal validity, it introduced selection bias with implications for real-world applicability. Additionally, volumetric estimations derived from CEUS-acquired linear dimensions may not fully capture true tumor geometry, particularly for subcentimeter nodules.

To further validate the clinical utility, future studies should employ three-dimensional CEUS reconstruction technology to improve volumetric measurement accuracy (30, 31), complemented with artificial intelligence (AI) -assisted ultrasound to characterize FTC-specific vascular patterns (32–34). Propose specific alternative biomarkers or adjusted algorithms should be proposed to determine true Tg levels in TgAb-positive cases. Furthermore, prospective multicenter studies are planned to further refine the diagnostic model, thereby contributing robust evidence toward the precision diagnostic criteria for thyroid follicular neoplasms.

## Conclusion

In summary, our study suggested that the Tg/Vol ratio > 7.412 as a clinically useful preoperative discriminator for FTC. The combined diagnostic model incorporating CEUS characteristics significantly improves diagnostic accuracy, mitigating risks of misdiagnosis in thyroid follicular neoplasms.

## Data Availability

The original contributions presented in the study are included in the article/**Supplementary Material**. Further inquiries can be directed to the corresponding author.
