# Supplementary material for: Thyroglobulin-to-tumor volume ratio combined with ultrasound features for diagnosing thyroid follicular neoplasms
Source: Front Endocrinol (Lausanne). 2025 Jul 10;16:1626766. doi: 10.3389/fendo.2025.1626766 (PMC12286834; doi:10.3389/fendo.2025.1626766)
Supplement: Supplementary file 1 [file DataSheet1.docx]

**Supplementary Materials** to Serum Thyroglobulin-to-Tumor Volume Ratio Combined with Contrast-Enhanced Ultrasound Features for Preoperative Diagnosis of Thyroid Follicular Neoplasms

**Supplementary Table 1. Comparison of CEUS Features Between FTA and FTC**

| CEUS Features | FTA (n = 377) | | FTC (n = 55) | | p-value |
| --- | --- | --- | --- | --- | --- |
| Tumor location |  |  |  |  | 0.158 |
| Left | 151 | (40.0%) | 21 | (38.2%) |  |
| Right | 193 | (51.2%) | 33 | (60.0%) |  |
| Bilateral and isthmus | 33 | (8.8%) | 1 | (1.8%) |  |
| Aspect ratio |  |  |  |  | 1.000 |
| < 1 | 354 | (93.9%) | 52 | (94.5%) |  |
| > 1 | 23 | (6.1%) | 3 | (5.5%) |  |
| Shape |  |  |  |  | 0.003^*^ |
| Regular | 139 | (36.9%) | 17 | (30.9%) |  |
| Relatively regular | 206 | (54.6%) | 25 | (45.5%) |  |
| Irregular | 32 | (8.5%) | 13 | (23.6%) |  |
| Margin |  |  |  |  | 0.020^*^ |
| Clear | 161 | (42.7%) | 21 | (38.2%) |  |
| Relatively clear | 181 | (48.0%) | 22 | (40.0%) |  |
| Unclear | 35 | (9.3%) | 12 | (21.8%) |  |
| Tumor composition |  |  |  |  | 0.172 |
| Solid | 196 | (52.0%) | 34 | (61.8%) |  |
| Cystic-solid | 181 | (48.0%) | 21 | (38.2%) |  |
| Adler blood flow grade |  |  |  |  | 0.773 |
| Grade 0 | 3 | (0.8%) | 1 | (1.8%) |  |
| Grade 1 | 25 | (6.6%) | 3 | (5.5%) |  |
| Grade 2 | 87 | (23.1%) | 15 | (27.3%) |  |
| Grade 3 | 262 | (69.5%) | 36 | (65.5%) |  |
| Calcification |  |  |  |  | < 0.001^*^ |
| None | 283 | (75.1%) | 37 | (67.3%) |  |
| Macrocalcification | 73 | (19.4%) | 7 | (12.7%) |  |
| Microcalcification | 21 | (5.6%) | 11 | (20.0%) |  |
| Relationship with surrounding tissue |  |  |  |  | < 0.001^*^ |
| No capsular involvement | 318 | (84.4%) | 33 | (60.0%) |  |
| Suspected capsular involvement | 54 | (14.3%) | 15 | (27.3%) |  |
| Capsular involvement | 5 | (1.3%) | 7 | (12.7%) |  |

FTA: Follicular thyroid adenoma; FTC: Follicular thyroid carcinoma

^*^ p-value < 0.05

**Supplementary Table 2. ROC Curve Analysis of FT3, Tg, and Tg/Vol Ratio in Predicting FTC**

|  | AUC | Cutoff Value | Sensitivity | Specificity |
| --- | --- | --- | --- | --- |
| Preoperative serum FT3 level | 0.589 | 5.045 pmol/L | 0.545 | 0.660 |
| Preoperative serum Tg level | 0.667 | 217.5 ug/L | 0.633 | 0.676 |
| Tg/Vol ratio | 0.664 | 7.412 | 0.796 | 0.516 |

FT3: Free triiodothyronine; Tg: Thyroglobulin; AUC: The area under the ROC Curve

**Supplementary Table 3. Binary Logistic Regression Analysis of Clinical Characteristics and CEUS Features in Predicting FTC** ^a^

|  | FTA (n = 377) | | FTC (n = 55) | | Univariate Logistic Regression | | | Multivariate Logistic Regression | | |
| --- | --- | --- | --- | --- | --- | --- | --- | --- | --- | --- |
|  |  |  |  |  | OR | (95% CI) | p-value | OR | (95% CI) | p-value |
| Male | 57 | (15.1%) | 23 | (41.8%) | 4.035 | (2.203 – 7.392) | < 0.001^*^ | 3.474 | (1.751 – 6.891) | < 0.001^*^ |
| Age (years) | 48 | (39 – 57) | 47 | (34 – 58) | 0.990 | (0.969 – 1.010) | 0.319 |  |  |  |
| BMI | 22.83 | (20.73 – 24.97) | 23.67 | (21.49 – 26.72) | 0.999 | (0.994 – 1.005) | 0.799 |  |  |  |
| Maximum tumor diameter (cm) | 4.60 | (3.60 – 5.70) | 4.40 | (3.80 – 5.70) | 0.973 | (0.702 – 1.349) | 0.869 |  |  |  |
| Tumor volume (cm³) | 18.35 | (9.67 – 37.27) | 21.22 | (11.47 – 38.61) | 1.003 | (0.989 – 1.018) | 0.636 |  |  |  |
| TgAb (IU/ml) ^b^ |  |  |  |  |  |  |  |  |  |  |
| ≤ 13.40 | 97 | (25.7%) | 15 | (27.3%) | 1.000 |  | - |  |  |  |
| 13.40 – 15.70 | 95 | (25.2%) | 15 | (27.3%) | 1.021 | (0.473 – 2.204) | 0.958 |  |  |  |
| 15.70 – 19.85 | 96 | (25.5%) | 6 | (10.9%) | 0.404 | (0.150 – 1.085) | 0.072 |  |  |  |
| > 19.85 | 89 | (23.6%) | 19 | (34.5%) | 1.381 | (0.662 – 2.881) | 0.390 |  |  |  |
| TPOAb (IU/L) ^c^ |  |  |  |  |  |  |  |  |  |  |
| ≤ 9.13 | 190 | (50.4%) | 27 | (49.1%) | 1.000 |  | - |  |  |  |
| 9.13 – 16.35 | 92 | (24.4%) | 15 | (27.3%) | 1.147 | (0.582 – 2.261) | 0.691 |  |  |  |
| > 16.35 | 95 | (25.2%) | 13 | (23.6%) | 0.963 | (0.475 – 1.951) | 0.917 |  |  |  |
| TSH (mIU/L) ^b^ |  |  |  |  |  |  |  |  |  |  |
| ≤ 1.19 | 95 | (25.2%) | 16 | (29.1%) | 1.000 |  | - |  |  |  |
| 1.19 – 1.89 | 93 | (24.7%) | 12 | (21.8%) | 0.766 | (0.344 – 1.707) | 0.515 |  |  |  |
| 1.89 – 2.87 | 94 | (24.9%) | 14 | (25.5%) | 0.884 | (0.409 – 1.913) | 0.755 |  |  |  |
| > 2.87 | 95 | (25.2%) | 13 | (23.6%) | 0.813 | (0.371 – 1.782) | 0.604 |  |  |  |
| FT3 (pmol/L) ^d^ |  |  |  |  |  |  |  |  |  |  |
| ≤ 5.045 | 249 | (66.0%) | 25 | (45.5%) | 1.000 |  | - |  |  |  |
| > 5.045 | 128 | (34.0%) | 30 | (54.5%) | 2.334 | (1.318 – 4.136) | 0.004^*^ | 1.708 | (0.894 – 3.263) | 0.105 |
| FT4 (pmol/L) ^b^ |  |  |  |  |  |  |  |  |  |  |
| ≤ 13.40 | 97 | (25.7%) | 14 | (25.5%) | 1.000 |  | - |  |  |  |
| 13.40 – 15.00 | 88 | (23.3%) | 17 | (30.9%) | 1.338 | (0.623 – 2.873) | 0.454 |  |  |  |
| 15.00 – 16.80 | 100 | (26.5%) | 14 | (25.5%) | 0.970 | (0.439 – 2.141) | 0.940 |  |  |  |
| > 16.80 | 92 | (24.4%) | 10 | (18.1%) | 0.753 | (0.319 – 1.780) | 0.518 |  |  |  |
| Shape |  |  |  |  |  |  |  |  |  |  |
| Regular | 139 | (36.9%) | 17 | (30.9%) | 1.000 |  | - | 1.000 |  | - |
| Relatively regular | 206 | (54.6%) | 25 | (45.5%) | 0.992 | (0.517 – 1.906) | 0.981 | 0.923 | (0.316 – 2.702) | 0.884 |
| Irregular | 32 | (8.5%) | 13 | (23.6%) | 3.322 | (1.466 – 7.527) | 0.004^*^ | 2.599 | (0.313 – 21.616) | 0.377 |
| Margin |  |  |  |  |  |  |  |  |  |  |
| Clear | 161 | (42.7%) | 21 | (38.2%) | 1.000 |  | - | 1.000 |  | - |
| Relatively clear | 181 | (48.0%) | 22 | (40.0%) | 0.932 | (0.932 – 0.494) | 0.827 | 0.966 | (0.334 – 2.795) | 0.949 |
| Unclear | 35 | (9.3%) | 12 | (21.8%) | 2.629 | (1.184 – 5.838) | 0.018^*^ | 0.649 | (0.082 – 5.109) | 0.681 |
| Calcification |  |  |  |  |  |  |  |  |  |  |
| None | 283 | (75.1%) | 37 | (67.3%) | 1.000 |  | - | 1.000 |  | - |
| Macrocalcification | 73 | (19.4%) | 7 | (12.7%) | 0.714 | (0.306 – 1.664) | 0.435 | 0.487 | (0.190 – 1.252) | 0.135 |
| Microcalcification | 21 | (5.6%) | 11 | (20.0%) | 3.546 | (1.553 – 8.098) | 0.003^*^ | 1.749 | (0.612 – 5.002) | 0.297 |
| Relationship with surrounding tissue |  |  |  |  |  |  |  |  |  |  |
| No capsular involvement | 318 | (84.4%) | 33 | (60.0%) | 1.000 |  | - | 1.000 |  | - |
| Suspected capsular involvement | 54 | (14.3%) | 15 | (27.3%) | 2.677 | (1.363 – 5.258) | 0.004^*^ | 1.914 | (0.880 – 4.162) | 0.101 |
| Capsular involvement | 5 | (1.3%) | 7 | (12.7%) | 13.491 | (4.054 – 44.895) | < 0.001^*^ | 9.958 | (2.453 – 40.424) | 0.001^*^ |

FTA: Follicular thyroid adenoma; FTC: Follicular thyroid carcinoma; TgAb: Thyroglobulin antibody; TPOAb: Thyroid peroxidase antibody; TRAb: Thyrotropin receptor antibody; TSH: Thyroid-stimulating hormone; FT3: Free triiodothyronine; FT4: Free thyroxine; OR: Odds ratio; CI: Confidence interval

^a^ Due to potential interference from elevated TgAb, analyses involving Tg and Tg/Vol ratio were performed only in TgAb-negative patients ( < 115.00 IU/mL). In the TRAb subgroup, TRAb levels were below the detection limit (0.8 IU/L) in more than half of the patients (n=323, 74.8%), so they were excluded from the analysis.

^b^ All patients were divided into 4 groups using the quartile method.

^c^ In the TPOAb subgroup, since 211 patients (48.8%) had TPOAb levels below the detection limit (9 IU/L), patients were divided into 3 groups based on the second and third quartiles.

^d^ In the FT3 subgroup, patients were divided into 2 groups using the optimal cutoff value (5.045 pmol/L) derived from univariate analysis.

^*^ p-value < 0.05

**
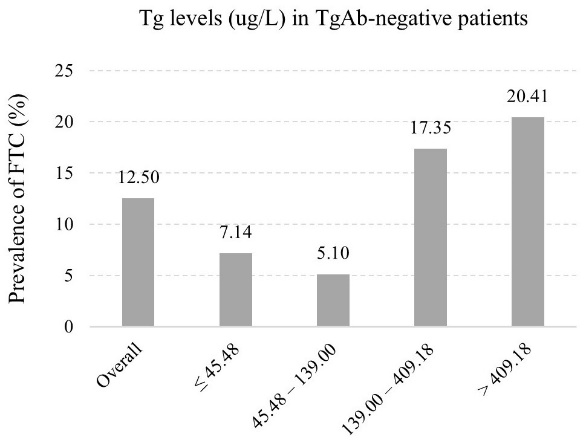
**

**Supplementary Figure 1.** Association between Tg levels and prevalence of TgAb-negative FTC. All TgAb-negative patients were divided into quartiles based on Tg levels, showing a significant increase in FTC prevalence with higher Tg levels (r = 0.176, p < 0.001).

**
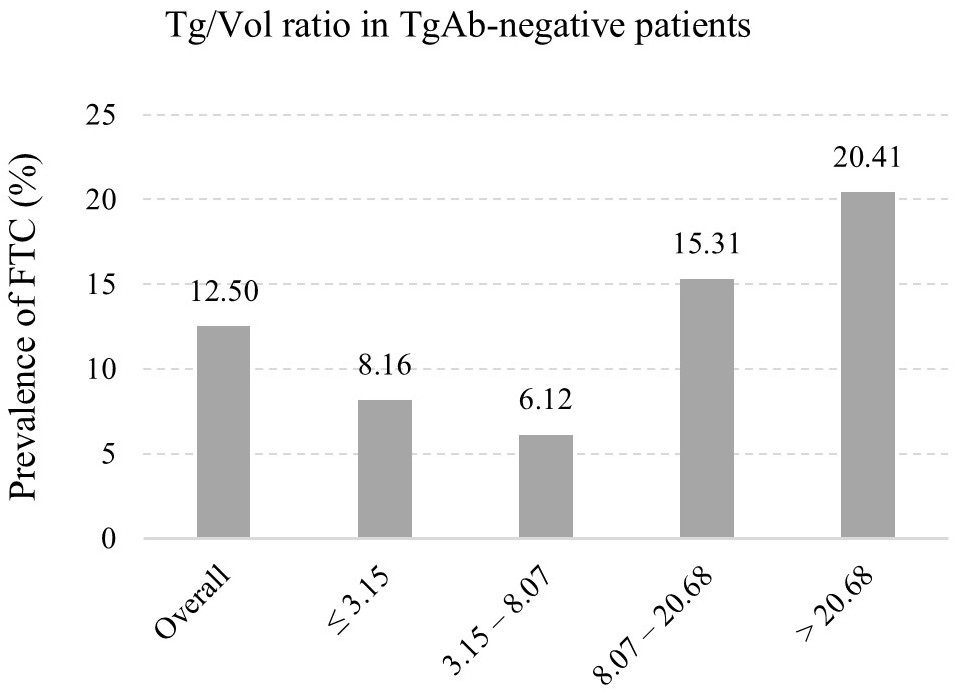
**

**Supplementary Figure 2.** Association between Tg/Vol ratio and prevalence of TgAb-negative FTC. All TgAb-negative patients were divided into quartiles based on Tg/Vol ratio, showing a significant increase in FTC prevalence with higher ratios (r = 0.155, p < 0.002).


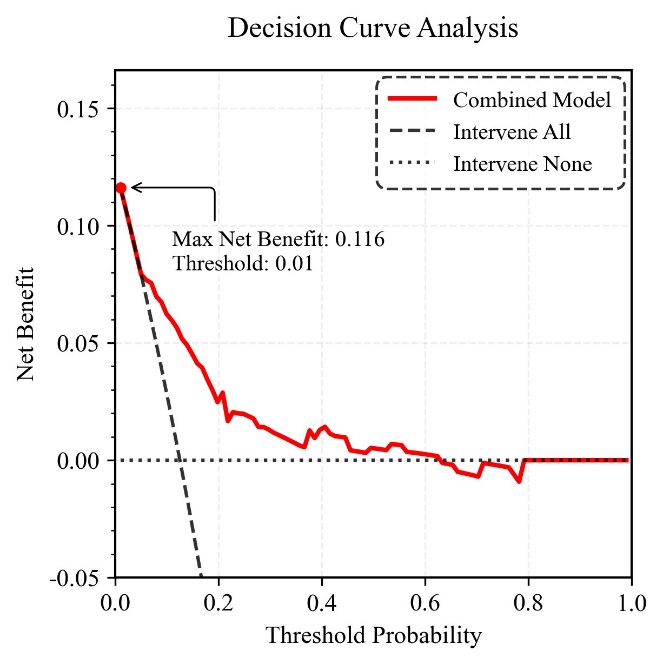


**Supplementary Figure 3.** Decision curve analysis of the combined diagnostic model. The combined diagnostic model was validated to exhibit explicit clinical value in the low-threshold interval (0.01-0.6), with the max net benefit reaching 0.116.


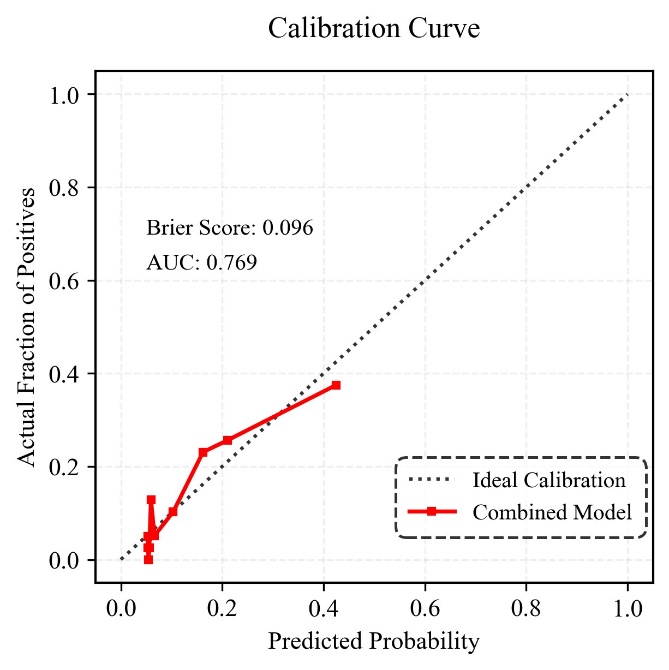


**Supplementary Figure 4.** Calibration curve of the combined diagnostic model. The combined diagnostic model was demonstrated excellent probabilistic calibration (Brier=0.096), and the curve's predictive probability range was constrained, lacking data points above 0.5, as the model rarely generated high predictive probabilities.


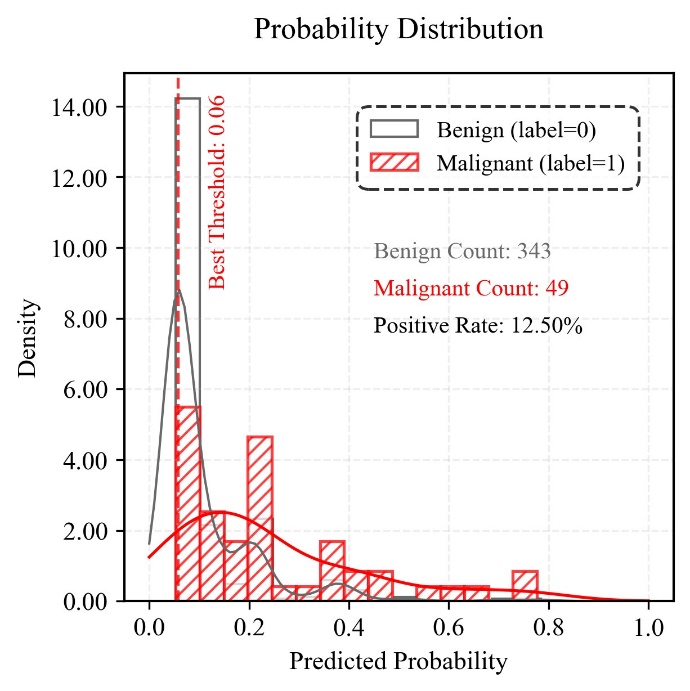


**Supplementary Figure 5.** Score distribution chart of the combined diagnostic model. The combined model effectively identified typical benign cases but struggled with atypical malignancies, yielding a mean malignant probability of 12.5%.
